# Plastic-Waste-Derived Char as an Additive for Epoxy Composite

**DOI:** 10.3390/ma16072602

**Published:** 2023-03-24

**Authors:** Seonho Lee, Yong Tae Kim, Kun-Yi Andrew Lin, Jechan Lee

**Affiliations:** 1 Department of Global Smart City, Sungkyunkwan University, 2066 Seobu-ro, Suwon 16419, Republic of Korea; 2 Chemical and Process Technology Division, Korea Research Institute of Chemical Technology, 141 Gajeong-ro, Daejeon 34114, Republic of Korea; 3 Innovation and Development Center of Sustainable Agriculture, Department of Environmental Engineering, National Chung Hsing University, 250 Kuo-Kuang Road, Taichung 402, Taiwan; 4 School of Civil, Architectural Engineering and Landscape Architecture, Sungkyunkwan University, 2066 Seobu-ro, Suwon 16419, Republic of Korea

**Keywords:** waste treatment, waste valorization, thermochemical process, epoxy resin

## Abstract

Tremendous amounts of plastic waste are generated daily. The indiscriminate disposal of plastic waste can cause serious global environmental issues, such as leakages of microplastics into the ecosystem. Thus, it is necessary to find a more sustainable way to reduce the volume of plastic waste by converting it into usable materials. Pyrolysis provides a sustainable solution for the production of carbonaceous materials (e.g., char). Plastic-waste-derived char can be used as an additive in epoxy composites to improve the properties and performance of neat epoxy resins. This review compiles relevant knowledge on the potential of additives for epoxy composites originating from plastic waste. It also highlights the potential of plastic-waste-derived char materials for use in materials in various industries.

## 1. Introduction

Plastic waste is complex and resistant to chemical and biological degradation [1].

Various practices involving landfilling, incineration, and mechanical and chemical recycling are usually employed to dispose of plastic waste [2,3,4]. Nevertheless, such practices have limitations associated with the economic returns, energy consumption, gas emissions, and quality of the resultant materials [1]. According to a recent report prepared by the Organization for Economic Cooperation and Development, the amount of plastics produced today is double of that produced 20 years ago; only 9% of the plastics are recycled, and the bulk is not properly managed. Mismanaged plastic waste is a serious source of pollution and toxins and has negative impacts on the environment [5]. The leakage of microplastics into ecosystems from industrial plastic pellets, tire wear, synthetic textiles, and road surface markings is a serious concern [6,7]. However, the development of plastic waste treatment technologies has been considerably slower than the growing demand for plastics.

Mechanical recycling is a typical method used for treating plastic waste. It suffers from a low recycling rate, poor quality of the recycled products, an inability to treat contaminated substances, and difficulties in treating plastic waste containing additives [8,9,10]. Chemical recycling is considered as a potential method to counteract the problems faced by mechanical recycling. Chemical recycling techniques involve depolymerization, solvolysis, thermochemical conversion processes. Depolymerization is used to recover monomers of mono plastics (i.e., only one type of plastic material used for manufacturing a whole product) such as plastic bottles and food trays [11,12]. The recovered monomers are repolymerized into new products [13]. Solvolysis involves the dissolution of a plastic product and is applied to certain types of plastic in the presence of solvents, e.g., expanded polystyrene in its monomer [14,15]. The thermochemical recycling—including also thermo-, photo-, and other oxidation processes—of plastic waste has recently attracted attention as an alternative and is considered an effective plastic waste conversion process [16,17,18,19,20,21]. The thermochemical conversion process can not only recover the chemical energy of plastic waste [22]; it can also convert plastic waste into original monomers, molecular intermediates for manufacturing other products, or high-value chemicals [23]. Moreover, the thermochemical treatment of plastic waste is environmentally benign [24] and economically feasible [25].

Among the various thermochemical plastic recycling methods, pyrolysis is particularly attractive for several reasons. Pyrolysis can treat mixed plastics, allowing for the handling of contaminated plastics [26]. A mobile system can be built because the scale can be efficiently reduced according to the size of the operation. This allows the system to be installed at sites with abundant feedstock [27]. Pyrolysis is a versatile process that anaerobically transforms waste feedstock (e.g., plastic waste) into products in various phases (e.g., gas, liquid, and solid) [28,29,30]. The product phase and yield can be readily controlled by varying the operational parameters (temperature, heating rate, residence time, reactor type, etc.) [31]. In addition, pyrolysis has a smaller environmental footprint than landfill, incineration, and gasification processes [32].

A solid-phase pyrolytic product (i.e., char) is obtained as a solid residue at the bottom of a pyrolizer as a result of the pyrolysis of plastic waste [33,34]. Char is a carbonaceous material that can be further upgraded to functional materials through pre- and/or post-treatment and is considered a sustainable and environmentally friendly material for a wide range of applications. For example, plastic-waste-derived char has shown promise as a soil conditioner [35], adsorbent [36], catalyst [37], electrode [38], and carbon sequestration material [39]. However, the use of plastic-waste-derived char in other applications, e.g., as an additive for an epoxy resin to make its composite, has gained much less interest, despite the need to develop sustainable alternatives.

Accordingly, the present review attempts to expand the application scope of plastic-waste-derived char by providing an overview of the latest information on the utilization of plastic-waste-derived char as an additive for epoxy resin. It is also expected to further enhance the significance of the pyrolysis process as a method for synthesizing a new class of sustainable materials from plastic waste.

## 2. Char Production from Plastic Waste

**Figure 1:**
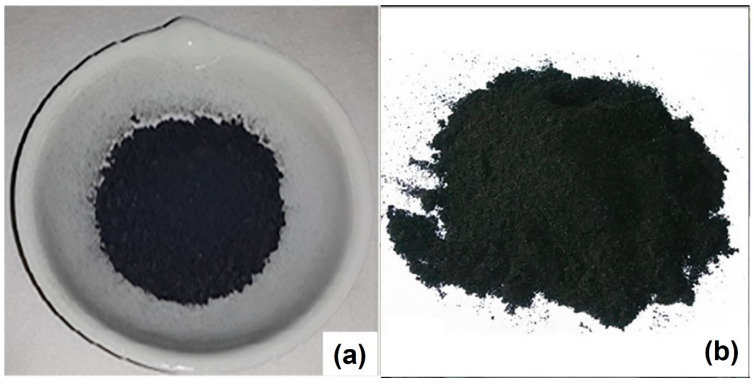
(**a**) Polypropylene-waste-derived char potentially used as an additive for producing epoxy composite. Reprinted from Sogancioglu et al. [41], Copyright (2019), with permission from Springer Nature. (**b**) Tire-waste-derived char potentially used as an additive for producing epoxy composite. Reprinted from Verma et al. [42], Copyright (2019), with permission from Wiley.

Char is a residual solid left in the pyrolizer at the end of the pyrolysis process. During pyrolysis, the plastics initially decompose into wax. The wax becomes a pyrolytic liquid that is further transformed into aromatic compounds and permanent gases. Ultimately, char is formed [40]. Figure 1 presents the physical appearances of polypropylene- (PP) and tire-waste-derived chars used as additives to produce epoxy composites [41,42]. The formation of char during the pyrolysis of plastic waste has been ascribed to secondary repolymerization reactions [43]. A heating rate lower than 80 °C min^−1^ is preferable for char production, i.e., to achieve a sufficiently long vapor residence time for more efficient secondary cracking reactions [44].

In general, the pyrolysis of plastics leads to lower char yields than the pyrolysis of organic carbonaceous substances, such as lignocellulosic biomass, and decreases with increasing pyrolysis temperature [45]. Therefore, to maximize the char yield from plastic waste, the pyrolysis of plastic waste must be conducted at temperatures lower than the typical pyrolysis temperatures for biomass (e.g., >300 °C). Table 1 summarizes the pyrolysis conditions under which chars are produced from various plastic wastes, yields of the pyrolytic products of the plastic wastes, and properties of the resultant chars. As summarized in Table 1, most pyrolysis processes aimed at producing char from plastic waste are conducted at temperatures lower than 300 °C. The char yields obtained from the plastic pyrolysis range from 2 to 18 wt%, which are highly associated with the kind of plastic waste. The char yield can be considered the char content in plastic waste that is potentially used as an additive for epoxy composite.

The pyrolysis of a feedstock with a higher fixed carbon content than typical plastics (e.g., tire waste) [46] could be conducted at typical pyrolysis temperatures aimed at char production. Plastic-waste-derived char tends to have a higher carbon content than biomass-derived char, primarily because plastic waste contains more carbon than biomass [47,48]. For instance, the carbon content of the plastic-waste-derived char potentially usable for producing epoxy composite must be at least approximately 75 wt%, as shown in Table 1. Furthermore, the chars made from plastic waste used as an additive for producing epoxy composite have a wide range of particle sizes, ranging from 10 to 70 μm, and the particle size can be further reduced to 50–70 nm via ball milling (Table 1).

## 3. Application of Plastic-Waste-Derived Char as an Additive for Epoxy Composite

**Figure 2:**
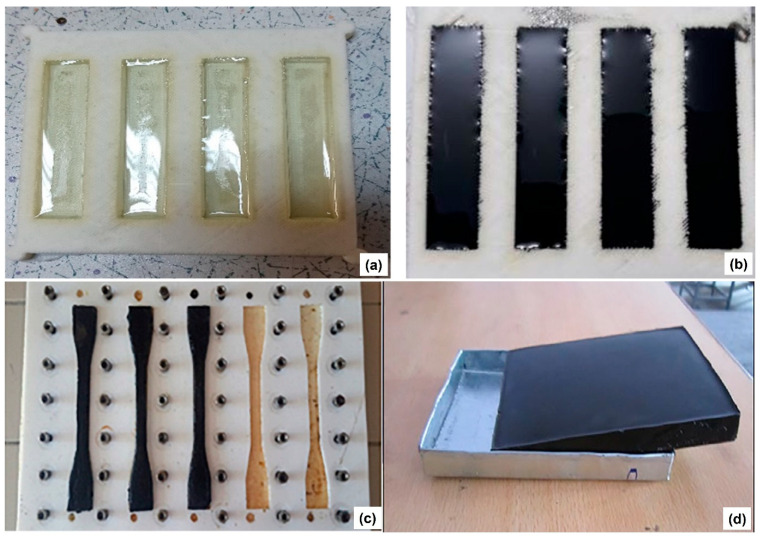
(**a**,**b**) Neat epoxy resin and epoxy composite made with a plastic-waste-derived char. Reprinted from Öner [53] and licensed under CC BY 4.0. (**c**) Epoxy composite made with polypropylene-waste-derived char. Reprinted from Sogancioglu et al. [41], Copyright (2019), with permission from Springer Nature. (**d**) Epoxy composite made with a tire-waste-derived char. Reprinted from Verma et al. [42], Copyright (2019), with permission from Wiley.

Studies have been conducted on the reuse of plastic-waste-derived char as an additive material for the preparation of polymeric composites with enhanced properties [58]. The representative results available in the literature are summarized in Table 2 and Table 3. Figure 2 shows examples of epoxy composites comprising different plastic-waste-derived chars. Sogancioglu et al. reported different epoxy composite materials made of polyethylene (PE) waste and PP-waste-derived char [41,50]. They also examined the possibility of using chars obtained from high-density PE (HDPE) and low-density PE (LDPE) waste as additive materials to prepare epoxy composites. Increasing the dosage of HDPE-waste- and LDPE-waste-derived char increased the electrical conductivity of the resultant composites with semiconductor structures (Nos. 3 and 4 in Table 2 and Table 3) [50]. The effect of the pyrolysis temperature at which the PP-waste-derived char was produced on the properties of the epoxy composite was also investigated between 300 °C and 700 °C. The results indicated that an epoxy composite material obtained with a PP-waste-derived char (10% dosage) produced at 300 °C exhibited the highest mechanical properties, such as tensile strength (99 MPa) and Young’s modulus (7.7 GPa), which are higher than those of a neat epoxy resin (No. 1 in Table 2 and Table 3) [41].

The characteristics of a composite made of epoxy resin and plastic-waste-derived char are highly dependent on several factors, including the char feedstock, the conditions at which the char is made, and char dosage, as those influence the carbon content and porosity of char. At comparable materials and conditions (Nos. 1–5 in Table 3), the pores present on char and the poor surface bonding of char particles lead to decreasing elongation at break. The immobilization of polymer chains in char results in high tensile strength. Young’s modulus and hardness are increased by the addition of char, most likely due to the carbon content in char. Electrical conductivity is also increased by adding char to neat epoxy resin, associated with aromatic structure in char structures.

In addition to polyolefin (e.g., PE and PP)-waste-derived char, poly(ethylene terephthalate) (PET)-waste-derived char has been employed as an additive material to produce epoxy composites [49,52]. The tensile strength, surface hardness, and Young’s modulus of the epoxy–PET-waste char composite were higher than those of a pure epoxy resin (No. 2 in Table 2 and Table 3) [49]. The impact of the pyrolysis temperature at which the PET waste-derived char was produced on the composite performance was also explored [52]. An epoxy composite made with a PET-waste-derived char additive produced at 300 °C had better properties (e.g., the tensile strength, elongation at break, conductivity, and surface hardness) than epoxy composites made with PET-waste-derived char additives produced at temperatures above 300 °C (No. 6 in Table 2 and Table 3) [52].

More recently, Wang et al. used carbon nanotubes grown on an alumina-supported iron catalyst via the pyrolysis of PP as a filler for an epoxy resin [59]. Ultrasonic dispersion was applied to achieve a uniform dispersion and to load the carbon nanotubes in the epoxy resin matrix. A PP-waste-derived carbon nanotube-based epoxy composite with a 2 wt% carbon nanotube loading exhibited superior mechanical properties in comparison with a neat epoxy resin, including a tensile strength of 37.3 MPa, fracture strength of ~112 Mpa, Young’s modulus of ~3780 Mpa, and fracture strain of ~6.3%. In other words, the addition of PP-waste-derived carbon nanotubes to the epoxy resin enhanced the toughness of the epoxy composite while retaining its stiffness. The predominant toughening mechanism for the PP-waste-derived carbon nanotube-based epoxy composite concerned the pull-out and bridging of the carbon nanotubes.

## 4. Summary and Outlook

The growing global demand for plastics is increasing the amount of generated plastic waste, causing serious environmental issues worldwide. The pyrolysis process is advantageous for reducing the volume of plastic waste and converting plastic waste into high-value products such as fuels (e.g., gas and liquid pyrolysates) and functional materials (e.g., char). The yield of each pyrolysate can be altered by controlling the pyrolysis conditions, such as the temperature, heating rate, and feedstock residence time. In particular, plastic-waste-derived char can be employed in the preparation of industrial materials as additives for epoxy resins to prepare epoxy composites. A plastic-waste-derived bitumen modifier and epoxy additive have shown potential as sustainable alternatives to a base bitumen and neat epoxy resin. Thus, the conversion of plastic waste into an additive for epoxy resin is a preferable option for mitigating the solid waste problem. Several research groups have demonstrated that plastic waste is a potential feedstock for producing industrial polymeric composites, providing a more eco-friendly approach than being discarded.

In the present review, recent outcomes achieved with char derived from different plastic wastes as potential additives for epoxy resins are introduced and discussed. The blending of conventional epoxy resin with plastic-waste-derived char leads to enhancing several properties such as tensile strength, Young’s modulus, hardness, and electrical conductivity. The extent of the enhancement is mainly associated with the kind of plastic used as the char feedstock and the char production conditions. However, the relationship and correlation between the composite characteristics and the char feedstock and synthesis conditions have not yet been fully elucidated. Accordingly, more studies on optimizing the characteristics of epoxy composite made of epoxy resin and plastic-waste-derived char need to be conducted.

Another issue is that direct comparisons of the available literature results are difficult. This is because the experiments have been conducted under different reaction conditions in different studies, and more importantly, the necessary experimental details were not always provided. Thus, it is hard to conclude that what kind of plastic and synthesis conditions are best for improving the mechanical properties of epoxy composite. To overcome this limitation, the methods or procedures for the synthesis of epoxy composites using plastic-waste-derived char should be standardized with a categorization of the plastic waste depending on its application.

Overall, the use of plastic-waste-derived char shows promise as an additive for preparing epoxy composites with enhanced properties. However, there are still limitations that need to be overcome in order to industrialize the applications of plastic-waste-derived char. With the technological developments in these approaches, the collection and transport of plastic waste should be considered to make the applications for plastic-waste-derived char more realistic.

## Figures and Tables

**Table 1:** Production of char from different plastic waste feedstocks: pyrolysis conditions, product yields, and char properties.

No.	Plastic Waste	Pyrolysis Conditions		Pyrolysate Yield (%)			Char Properties			Ref.
		*T* (°C)	Heating Rate (°C min^−1^)	Gas	Oil	Char	Surface Area (m^2^ g^−1^)	Particle Size (µm)	Elemental Composition (wt%)	
1	Polypropylene (PP) waste	300–700	5	17.7–22.8	75.1–79.6	2.2–2.7	13.5–22.0	-	-	[41]
2	Polyethylene terephthalate (PET) waste	450	-	-	-	-	-	<63	C = 74.7, O = 21.8, K = 2.4, Mg = 0.3, Ca = 0.8	[49]
3	High-density polyethylene (HDPE) waste	300–700	5	9.1–14	83.8–88.5	2.1–2.3	-	<63	-	[50]
4	Low-density polyethylene (LDPE) waste	300–700	5	11.5–21.4	72.9–78.4	6.4–10.1	-	<63	-	[50]
5	PET waste	300–700	-	-	-	-	-	<63	-	[51]
6	PET waste	300–700	-	-	-	-	-	~63	-	[52]
7	Tire waste	-	-	-	-	-	-	-	-	[53]
8	Tire waste	~525	-	-	-	-	30.4	<45	C = 79.2, S = 1.5	[54]
9	Tire waste	~315	-	-	-	-	-	50–70 nm (8-h milling at >2500 rpm)	C = 86.0, O = 5.4, S = 2.3, Zn = 5.1, Al = 0.4, Si = 0.7	[42]
10	Food packaging plastic waste	600	25	-	-	18.6	-	10–15	-	[55]
11	Expanded polystyrene (PS) foam waste	530 ^a^	10	-	-	-	2712	-	C = 94.4, O = 3.8, H = 0.2, N = 0.2	[56]
12	PS waste + Eucalyptus biomass ^b^	300–550	10	-	-	18–38	-	-	Fixed C = 4.5–34.2	[57]

^a^ After pyrolysis, the char was activated at 800 °C for 1 h and treated with 10% HCl; ^b^ PS waste/biomass ratio = 1/2 or 1/3 (*w*/*w*).

**Table 2:** Synthesis methods of epoxy composite using the plastic-waste-derived char as an additive.

No. (Same as No. in Table 1)	Epoxy Resin	Char Feedstock	Additive Dosage (%)		Condition for Epoxy Composite Synthesis			Ref.
			Char	Other Supplement (Dosage)	Preparation	Degassing	Curing	
1	Not specified	PP waste	10–50	Hardener (30) Accelerator (1)	Stirred under 2000 rpm for 3 h	40 °C for 1 h	40–120 °C for 3 d	[41]
2	NPEL-128	PET waste	5–30	Epamine PC17 as hardener (30) tris-DMP as accelerator (1)	Stirred under 1000 rpm for 3 h Ultrasonicated at 60 °C for 1 h	RT for 1 h	40 °C for 1 d 60–120 °C for 2 d	[49]
3	NPEK-114	HDPE waste	10–50	Epamine PC17 as hardener (30) tris-DMP as accelerator (1)	Stirred under 2000 rpm at room temperature for 3 h Ultrasonicated at 60 °C for 1 h	40 °C for 1 h	40 °C for 1 d 60–120 °C for 2 d	[50]
4	NPEK-114	LDPE waste	10–50	Epamine PC17 as hardener (30) tris-DMP as accelerator (1)	Stirred under 2000 rpm at room temperature for 3 h Ultrasonicated at 60 °C for 1 h	40 °C for 1 h	40 °C for 1 d 60–120 °C for 2 d	[50]
5	NPEK-114	PET waste	10–50	Epamine PC17 as hardener (30) tris-DMP as accelerator (1)	Stirred under 1000 rpm for 3 h	RT for 1 h	40 °C for 1 d 120 °C for 2 d	[51]
6	NPEK-114	PET waste	10–50	Hardener (30) Accelerator (1)	Stirred under 1000 rpm for 3 h	RT for 1 h	40 °C for 1 d 60–120 °C for 2 d	[52]
7	DTE-1200	Tire waste	-	DTS-1151 as hardener	After adding char, mixing for 10 min Adding hardener, stirred under 500 rpm for 5 min	-	-	[53]
8	Polires-188	Tire waste	3	Cardolite NC-562 as hardener	Mixed with char in acetone for 10 min Mixed with hardener for 5 min	RT for 2 h	80 °C for 2 d	[54]
9	CY-230	Tire waste	5–15	HY-951 as hardener (9)	100 °C and 200 revolutions for 1 h Heated under microwave at 80–100 °C for 1 h	Cooled down to 35–45 °C Mixed with hardener for 5 min Solidified for 1 d	110 °C for 3 h under vacuum	[42]
10	MGS RIMR-135	Food packaging plastic waste	0.25–1	MGS RIMH-1366 as hardener (30)	Char dispersion in acetone at RT for 1 h Mixed with char at 25 °C for 3 h Mixing at 50 °C for 30 min	After adding hardener, mixed for 15 min Exposed to vacuum infiltration for 15 min	90 °C for 8 h 85 °C for 7 h under IR	[55]

tris-DMP: 2,4,6-tris(dimethylaminomethyl)phenol; RT: room temperature; IR: infrared radiation

**Table 3:** Comparing representative characteristics of neat epoxy and epoxy composites made from epoxy resin and plastic-waste-derived char.

No. (Same as No. in Table 1)	Epoxy Composite	Elongation at Break (%)		Tensile Strength (MPa)		Young’s Modulus (GPa)		Hardness (Shore D, Otherwise Mentioned)		Electrical Conductivity (S cm^−1^)		Ref.
		Neat Epoxy	Composite	Neat Epoxy	Composite	Neat Epoxy	Composite	Neat Epoxy	Composite	Neat Epoxy	Composite	
1	PP waste char/epoxy resin ^a^	0.71	0.62	85	99	6.2	7.7	80	83	10^−14^	4.2 × 10^−7^	[41]
2	PET waste char/NPEL-128 ^b^	0.53	0.52	0.47	0.59	82	110.7	83	87.6	10^−14^	2.0 × 10^−5^	[49]
3	HDPE waste char/NPEK-114 ^a^	0.52	0.55	62	72	-	-	80	85	8.4 × 10^−13^	4.7 × 10^−5^	[50]
4	LDPE waste char/NPEK-114 ^a^	0.52	0.25	62	42	-	-	80	73	8.4 × 10^−13^	4.3 × 10^−8^	[50]
5	PET waste char/NPEK-114 ^a^	0.72	0.69	86	97	6.2	9.4	-	-	-	-	[51]
6	PET waste char/NPEK-114 ^a^	-	-	62	98	-	-	80	85	-	7.98 × 10^−5^	[52]
7	Tire waste char/DTE-1200 ^c^	-	-	-	-	-	-	-	-	-	-	[53]
8	Tire waste char/Polires-188	-	-	-	-	6.7	3.0	415.9 MPa	165.7 MPa	-	-	[54]
9	Tire waste char/CY-230 ^d^	7.1	7.6	33.8	34.6	0.63	0.74	130 HRL	140.7 HRL	1.96 × 10^−3^	2.4 × 10^−3^	[42]
10	Food packaging plastic waste char/MGS RIMR 135 ^e^	2.3	1.8	188.2	176.4	6.58	7.79	-	-	-	-	[55]

^a^ Char made at 300 °C and char dosage of 30%; ^b^ Char dosage of 15%; ^c^ Char dosate of 1 wt%; Flexural strength = 69.4 MPa (neat epoxy) vs. 77.5 MPa (composite); Glass transition temperature = 59.5 °C (neat epoxy) vs. 61.4 °C (composite); ^d^ Char made at ~315 °C and char dosage of 15%; ^e^ Char dosage of 1 wt%

## Data Availability

Data available on reasonable request from the corresponding author.
